# Longitudinal healthcare utilization among traumatic spinal cord injury patients: a 20 year retrospective study using population-based data

**DOI:** 10.1186/s12913-025-13895-z

**Published:** 2025-12-15

**Authors:** Michael Bond, Aidan Beresford, Vanessa K. Noonan, Naama Rotem-Kohavi, Brian K. Kwon, Guiping Liu, Jason M. Sutherland

**Affiliations:** 1 Centre for Health Services and Policy Research, University of British Columbia, Vancouver, Canada; 2 Praxis Spinal Cord Institute, Vancouver, BC Canada; 3 Combined Neurosurgery and Orthopaedic Spine Program, University of British Columbia, Vancouver, B.C Canada

**Keywords:** Traumatic spinal cord injury, Health care utilization, Primary care, Administrative data

## Abstract

**Background:**

Patients with traumatic spinal cord injury (TSCI) experience the healthcare system in a heterogeneous fashion after initial injury. This study performs a retrospective analysis of administrative data to identify patterns of longitudinal healthcare utilization among patients with TSCI in British Columbia, Canada, up to 20 years after initial hospitalization.

**Methods:**

Using population-based administrative databases, adult patients with incident TSCIs were identified between January 2001 and December 2021. Population-based healthcare administrative and demographic data were used to determine physician services (primary care and specialist), hospital admissions (elective surgical, medical, and emergency department), and clinical information. Descriptive summaries measuring healthcare utilization per person year were calculated. Average utilization calculated in person years since the time of injury was compared between those under 65 years of age and those who were over 65, and based on the level of injury (cervical vs. thoracic/lumbar). Latent-class analysis identified characteristics associated with high healthcare utilization.

**Results:**

The cohort included 4132 patients with an incident TSCI. On average, the patients had 18.9 primary care provider (PCP) visits per person year after their injury occurred. Patients had 13.9 specialist visits per person year, of which the most common was with a neurologist. The average rate of hospital admission for all patients was 1.4 visits per year, and emergency department encounters occurred on average were 0.7 visits per year. Patients 65 and over and those with cervical injuries consistently utilized more healthcare resources compared to younger patients and those with thoracic/lumbar injuries (*p* < 0.001). Latent class modelling found that the highest healthcare utilization was among those with cervical spinal cord injuries and who lived in an urban area.

**Conclusions:**

Patients with TSCI had heterogeneous patterns of primary and specialist healthcare utilization up to 20 years after injury. Further analysis revealed that patients who had had cervical injuries and resided in urban centres accessed healthcare resources more frequently.

**Supplementary Information:**

The online version contains supplementary material available at 10.1186/s12913-025-13895-z.

## Background

Traumatic spinal cord injury (TSCI) is a significant event that has long-lasting implications for the individual and their use of healthcare services and resources [1]. TSCIs can result in irreversible damage to sensory and motor function and lead to medical complications that impact quality of life [2, 3]. Although TSCIs are rare events, with a global rate of 1 in 23 million [4], the demand for healthcare services among this population is high due to the complex nature of the injury requiring long-term care and support. Higher healthcare utilization can create a significant economic impact on healthcare systems and patients, in addition to the need for comprehensive acute and rehabilitation-based care immediately after initial injury [5–8]. Further, the demographics of TSCI are shifting, and there is an increasing incidence of TSCIs among the elderly population due to low-energy falls [9, 10]. The increased rate of elderly patients suffering TSCI from low-energy falls may cause new system-level challenges, given that elderly TSCI patients are more likely to have multiple medical issues and chronic disease diagnoses prior to their spinal cord injury, requiring more specialized and complex care [11, 12]. There is a paucity of information regarding healthcare utilization among those with TSCI.

Management of TSCI after acute hospitalization involves transition into community-based care, and for many TSCI patients, there is no clear clinical pathway that provides access to out-of-hospital services [13–15]. In many healthcare systems, delivery of healthcare to TSCI patients is provided through a variety of services, settings and providers, causing difficulties with navigating appropriate care [16]. The transition to outpatient care is a particular challenge in Canada due to a lack of rehabilitation services provided through public insurance to assist patients in post-injury rehabilitation, often creating a patchwork of services provided to those with TSCI [17]. Given this, how and where TSCI patients access healthcare services is poorly understood. This lack of insight makes it difficult for clinicians, patients, and policymakers to address gaps in care, anticipate healthcare staffing needs, and develop clinical care pathways that aim to improve the health of TSCI patients across the lifespan.

The objective of this study is to describe patterns of longitudinal healthcare utilization, including primary care physicians (PCP), specialist consultations, emergency department (ED) visits, and all-cause hospital admissions among TSCI patients in the province of British Columbia (B.C.), Canada, using population-based data. A secondary objective is to determine which patient or system-level characteristics are associated with higher rates of utilization. This study’s findings are important to patients, clinicians and policymakers as they will illustrate the constellation of patients’ healthcare utilization following TSCI and identify gaps where access to healthcare services can be improved.

## Methods

### Study design and population

This study was based on retrospective data from a population-based observational cohort of adults having had a TSCI in the province of B.C. between January 2001 and December 2021. This study utilized population-based hospital and physician-based administrative data routinely collected and maintained for research purposes by Population Data B.C [18]. Population Data B.C. provides research access to linkable, longitudinal, de-identified patient-level datasets for all publicly insured individuals in the province. Data was extracted from several sources, including the Canadian Institute for Health Information Discharge Abstract Database (DAD), B.C. Provincial Vital Statistics, the National Ambulatory Care Reporting System (NACRS), and the Medical Services Plan (MSP) Payment Information File, which includes physician billing data [19–23]. The protocol for linkage has been described elsewhere [24]. Patients not publicly funded through MSP were not included in this study, this included temporary visitors and tourists, and federally funded groups such as those active in the Canadian Armed Forces or incarcerated in a federal penitentiary.

The cohort was created based on identifying hospital discharge records with an International Classification of Diseases 10-CA diagnosis code (see Appendix 1 for list of codes) for TSCI. The cohort included all patients hospitalized for acute cervical, thoracic, lumbar, sacral, or cauda equina TSCIs in B.C. over the study period. The TSCI case definition was validated by mapping ICD-10-CA codes to International Standards for Neurological Classification of Spinal Cord Injury (ISNCSCI) descriptions of TSCI by level and severity and has been described elsewhere [24]. Patients were excluded if they died during initial hospitalization or were under the age of 18 at the time of injury. All patients were continuously insured throughout the study period through provincially funded and publicly available plans. For cohort members, administrative and clinical data were deterministically linked and anonymous to the study team. This unique linked data has been described elsewhere [24]. Ethics approval for analysis of population-based data was obtained from the University of British Columbia Research Ethics Board (REB#: H22-02696).

### Demographic and clinical variables

The demographic and clinical variables were identified from the initial TSCI injury hospitalization, and included age (grouped in categories < 35, 35–64, and *≥* 65 years old), sex (male, female), Charlson Comorbidity Index (CCI), mechanism of injury (falls, transport, other), and level of injury (cervical vs. thoracic/lumbar), presence of traumatic brain injury (TBI), and Injury Severity Score (ISS) [25]. Sociodemographic status was determined using the Quintile of Adjusted Income per person Equivalent (QAIPPE) [26], which is a measure of neighbourhood income adjusted for household size determined by Statistics Canada. Place of residence was determined as rural or urban based on the first 3 characters in their postal code [22].

### Outcomes

The main outcome of this study was the rate of utilization of physician-based services, as identified through physician billing data and expressed as visits per person year averaged from the time of injury to the end of the study period in December 2021. Physician-based services included PCP visits and specialist visits (including neurologists, physical medicine and rehabilitation, orthopaedic surgeons, neurosurgeons, urologists, and psychiatrists). Hospital-based care was identified using the DAD, identifying patients admitted to the hospital for acute medical issues, emergency surgeries or having had elective surgical procedures. For all ED visits, physician billing data (MSP) was used to identify the service date of the physician consultation. Physician visits or multiple billings on the same day were counted as a single encounter.

### Statistical analysis

Demographic and clinical information were summarized using means and standard deviations for continuous variables and counts for categorical variables. Health care visits were reported in rates per person year for all participants to capture all observed time for each patient after discharge from acute care. Physician visits per person year in the outpatient setting were calculated for primary care and specialist interactions during the study period. Inpatient hospitalizations, ED visits, and elective surgical procedures were summarized similarly. Comparisons between the rates of visits per person year were performed using level of injury (cervical and thoracic/lumbar injury) and age category (*≥* 65 years old and. < 65 years old). Per person year rates and confidence intervals were calculated using Poisson regression. Overdispersion was addressed by applying a scaling parameter to the estimated variance. Statistical significance was set a *p* < 0.05, and all statistical analysis was performed using SAS v9.4.

To identify clinical and demographic characteristics of patients with TSCI who access healthcare services most frequently, a finite mixture model latent class model was fit using maximum likelihood. In this analysis, the observed variables included were per patient-year visits for primary care, specialists’ services (urology, neurology, orthopedic, neurosurgery, physiatry, and psychiatry) and per-patient-year visits for elective surgeries. Other variables included in the model were patient age (< 65 or *≥* 65 years), sex, CCI, QAIPPE, and place of residence (urban vs. rural). A multi-group latent class analysis used level of injury to identify two distinct groups. Further refinement of the classes selected was accomplished using the Akaike Information Criterion (AIC) and Bayesian Information Criterion (BIC). A step-wise approach was taken to add additional latent classes into the model; based on the fit criteria the 4-class model described variability in the data the best. Latent class analysis was performed using Mplus V8.3 [27]. Nonparametric bootstrap techniques were applied to address non-normality of the data.

## Results

Over the course of this 22-year study period, 4132 patients met the cohort’s inclusion criteria. The mean age was 55.1 years (SD 19.64), and 2973 (72.0%) were male. In this cohort, 1661 (40.2%) patients had thoracic/lumbar Injuries, and 2471 (59.8%) had cervical spinal cord injuries. 1762 (42.6%) of patients received their injury from a fall. (See Table 1 for full demographic and clinical details).

**Table 1:** Summary presentation of the TSCI cohort

Characteristic	Total (*N* = 4132)
Age (SD)	55.1 (19.2)
Male	2973 (72.0%)
Charlson Comorbidity Index (SD)	0.3 (0.9)
QAIPPE	
1- Lowest	985 (23.8%)
2	846 (20.5%)
3	801 (19.4%)
4	726 (17.6%)
5	689 (16.7%)
Missing	85 (2.1%)
Level of Injury	
Cervical (C1-C8)	2471 (59.8%)
Thoracic (T1-T12)	660 (16.0%)
Lumbar (L1-L5)	947 (22.9%)
Sacral/Cauda Equina (S1-S5)	54 (1.3%)
Injury Severity Score	24.2 (13.6)
Traumatic Brain Injury	
Yes	543 (13.1%)
No	3589 (86.9%)
Mechanism of Injury	
Falls	1762 (42.6%)
Transport	1241 (30.0%)
Others	1129 (27.4%)

Demographic and clinical characteristics and stratified by level of injury (Thoracic / Lumbar Injury vs. Cervical Injury)

The study found that on average, patients with TSCI were seen by a PCP 18.9 (18.0 to 19.9 95% C.I.) visits per patient-year since the time of their injury. All specialist visits averaged 13.9 (13.1 to 14.7 95% C.I.) visits per patient-year, with neurology having had 6.4 (6.0 to 6.7 95% C.I.) visits per patient-year, physiatry 5.3 (4.8 to 5.7 95% C.I.) visits per patient-year, and psychiatry with 3.1 (2.8 to 3.4 95% C.I.) visits per patient-year. Further, it was identified that patients with cervical spinal cord injuries and those 65 and over visited both PCPs and specialists more frequently compared with those under 65 (*p* < 0.001). Patients with TSCI were admitted to the hospital 1.4 (1.3 to 1.5 95% C.I.) times per patient-year, had 0.6 (0.6 to 0.7 95% C.I.) visits per patient-year to the ED and had 0.3 (0.3 to 0.4 95% C.I.) elective surgical procedures per patient-year. Those with cervical injuries or aged 65 and over were hospitalized most frequently. See Table 2 for further details.

**Table 2:** Rates of primary Care, Specialist, hospital admissions, elective surgeries, and ED visits per patient-year. The Poisson distribution was used to Establish 95% confidence intervals

	Total		Thoracic / Lumbar Injury		Cervical Injury		Age < 65		Age *≥* 65	
	Rate	CI	Rate	CI	Rate	CI	Rate	CI	Rate	CI
Primary Care	18.9	(18.0 to 19.9)	17.1	(15.7 to 18.4)	20.3	(18.9 to 21.6)	13.8	(13.1 to 14.4)	30.1	(27.5 to 32.7)
Specialist										
Urology	1.4	(1.3 to 1.4)	1.3	(1.1 to 1.4)	1.4	(1.3 to 1.6)	1.1	(1.1 to 1.2)	1.9	(1.7 to 2.2)
Neurology	6.4	(6.0 to 6.7)	4.8	(4.4 to 5.3)	7.4	(6.9 to 8.0)	5.3	(4.9 to 5.7)	8.6	(7.8 to 9.4)
Orthopaedic	1.2	(1.1 to 1.3)	1.1	(1.0 to 1.2)	1.3	(1.1 to 1.5)	1.0	(0.9 to 1.1)	1.8	(1.4 to 2.2)
Neurosurgery	1.5	(1.3 to 1.8)	1.2	(1.0 to 1.3)	1.8	(1.4 to 2.2)	1.0	(0.8 to 1.2)	2.6	(2.0 to 3.2)
Physiatry	5.3	(4.8 to 5.7)	3.8	(3.2 to 4.3)	6.1	(5.5 to 6.7)	4.7	(4.2 to 5.2)	6.7	(5.8 to 7.7)
Psychiatry	3.1	(2.8 to 3.4)	2.6	(2.2 to 3.0)	3.5	(3.0 to 4.0)	2.8	(2.4 to 3.1)	3.9	(3.2 to 4.6)
Hospital Admissions	1.4	(1.3 to 1.5)	1.2	(1.1 to 1.3)	1.5	(1.4 to 1.7)	1.1	(1.0 to 1.1)	2.1	(1.9 to 2.3)
Elective Surgeries	0.3	(0.3 to 0.4)	0.4	(0.3 to 0.4)	0.7	(0.6 to 0.7)	0.3	(0.3 to 0.4)	0.5	(0.4 to 0.5)
Emergency Department	0.6	(0.6 to 0.7)	0.6	(0.6 to 0.6)	0.3	(0.3 to 0.3)	0.5	(0.5 to 0.6)	0.9	(0.8 to 1.0)

**Fig. 1:**
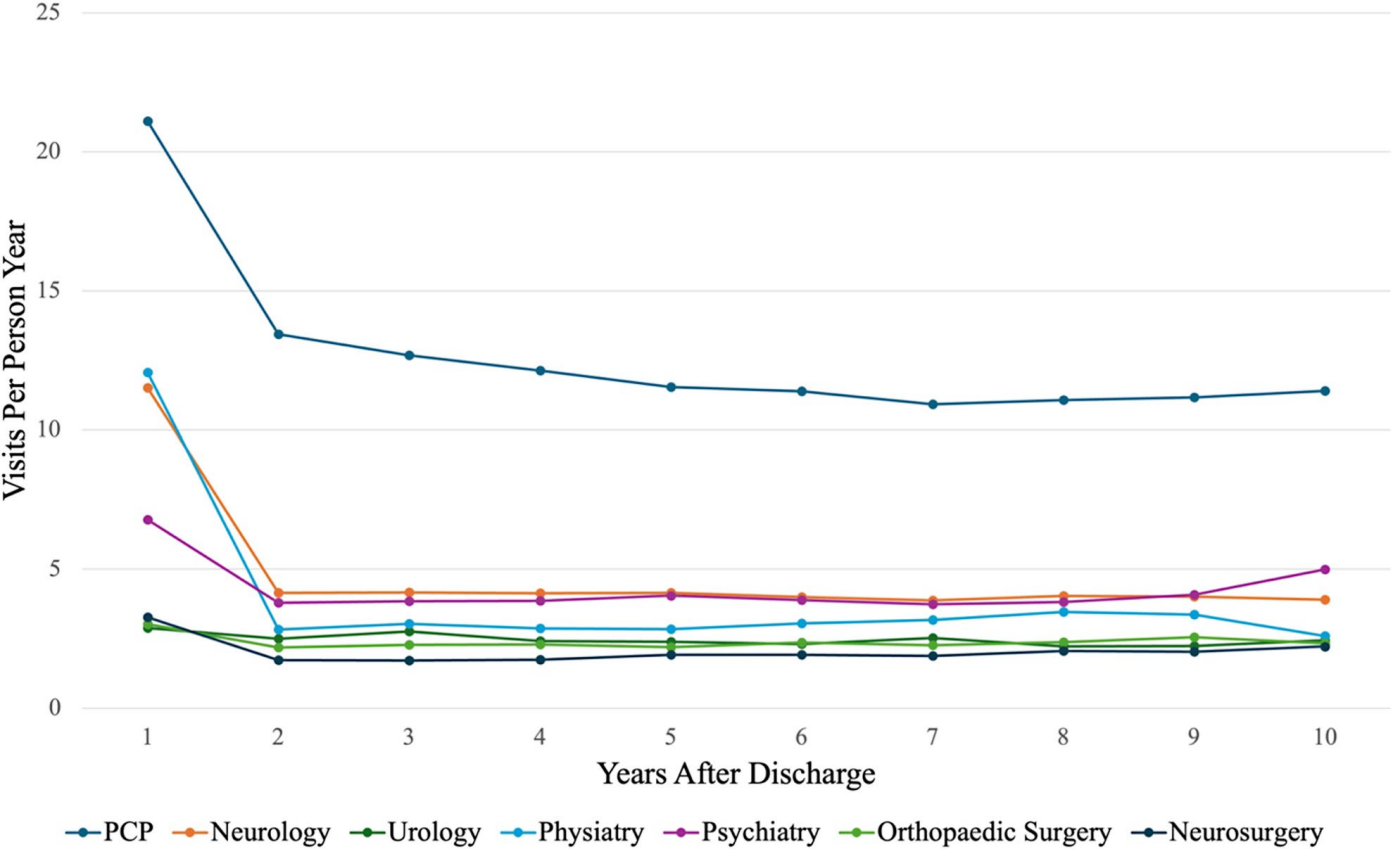
Physician visits over 10 years after discharge from acute care for all TSCI patients

Figure 1 illustrates the trends in healthcare utilization for physician services in the first 10 years after discharge for TSCI. This figure demonstrates that cohort members had the highest healthcare utilization in the first year following their discharge from acute care. Rates of PCP and specialist visits decreased after the first year and remained stable over time.

Latent class analysis found four mutually exclusive groups based on healthcare utilization patterns. These four patterns were divided by level of the spinal cord injury (cervical spine vs. thoracic/lumbar injury) and the location of residence (rural vs. urban). Those who had cervical spinal cord injuries and were living in urban centers had the highest rates of healthcare utilization. Those who had thoracic/lumbar injuries and lived in rural locations at the time of their injury had the lowest rates of healthcare utilization. See Table 3 for details.

**Table 3:** Latent class analysis model and visits estimates with standard errors, including four classes; class 1: Thoracic/Lumbar – Urban, class 2: Thoracic/Lumbar – Rural, class 3: Cervical – Urban, class 4: Cervical – Rural

	Class One	*N* = 1355	Class Two	*N* = 266	Class Three	*N* = 1886	Class Four	*N* = 369
	Estimate	Standard Error	Estimate	Standard Error	Estimate	Standard Error	Estimate	Standard Error
Primary Care	17.64	0.72	13.99	1.33	20.75	0.49	16.41	1.60
Specialist (Total)	11.40	0.67	6.75	0.92	17.20	0.45	12.03	1.65
Urology	0.67	0.03	2.47	0.42	7.46	0.21	4.92	0.77
Neurology	4.93	0.32	0.74	0.10	0.79	0.09	0.50	0.13
Orthopaedic	0.74	0.03	0.81	0.08	1.26	0.09	1.12	0.16
Neurosurgery	0.85	0.07	1.23	0.34	4.16	0.19	3.12	0.61
Physiatry	2.07	0.26	1.05	0.08	2.77	0.19	1.79	0.34
Psychiatry	2.16	0.09	6.76	0.92	17.20	0.45	12.00	1.65
Hospital Admissions	1.20	0.04	1.15	0.10	1.57	0.07	1.33	0.28
Emergency Department	0.57	0.06	0.24	0.07	0.61	0.04	0.34	0.07
Elective Surgeries	0.35	0.02	0.36	0.03	0.27	0.01	0.21	0.02

## Discussion

This study provided an overview of healthcare utilization among TSCI patients. Leveraging the study setting’s single-payer healthcare system, the study provides insights regarding utilization of primary care, specialist consultations, ED visits, and all-cause hospital admissions.

Primary care is an important component in the care of patients outside of the hospital setting and helps patients navigate the health care system to access the care they need. This is especially important for patients who have complex medical requirements, like those with TSCI [28, 29]. This is reflected in the results of this study in that PCP visits were the most frequent type of physician encounter, averaging 18.9 visits per person year after their initial injury. In comparison to the general population, where a recent study by Lavergne et al. (2022) found that PCP visits averaged between 3.6 and 4.7 visits per person per year using similar data. Using the Canadian Community Health Survey, stroke survivors tended to have on average 4.9 visits per year with their PCP [30]. In this study, PCP continue to be a significant contact point for patients during their life course after injury, with much higher rates of visits on average when compared with the general population and similar conditions. This increased utilization of primary care services extended far beyond other injuries, including brain injury and pelvic fracture [31].

PCPs can manage the majority of general medical issues; however, when further input is needed for more complex issues, patients in Canada’s gatekeeper model are then referred to community or hospital-based specialty physicians, such as neurologists, urologists, spinal surgeons (orthopaedic or neurosurgical), and physical and rehabilitation medical specialists [28]. This study found that TSCI patients received specialized care most frequently from neurologists, physiatrists, and psychiatrists. Specialist care represented a significant proportion of the TSCI’s clinical activity, with 13.9 visits per person year in the first year post-discharge from acute care. Specialists contribute essential expertise in managing TSCI-related issues and guiding ongoing rehabilitation. Coordinating care between multiple physicians is often challenging and complex due to the varied needs of TSCI patients [32, 33]. Ensuring appropriate communication between the PCP and specialists to ensure optimal care is essential to this relationship. To streamline this process and enhance coordination, there has been a recognition that a multidisciplinary approach to care for patients with TSCI is optimal; however, it is often difficult to achieve in complex health systems [34]. Studies have demonstrated that these models of care, including interactions between multiple specialists, allied health rehabilitation specialists, and PCP, are essential to avoid gaps in care and reduce complications [35]. To help support PCP management of TSCI, specialists should ensure they provide PCPs with detailed guidance and instruction on how to manage more complex issues.

The latent class analysis revealed that patients who had cervical spinal cord injuries had a greater number of encounters with the healthcare system, including PCP, specialist, hospital, and ED visits. This is most certainly the result of the more diffuse perturbations across multiple physiologic systems in those with cervical-level injuries as compared to thoracic-level injuries (tetraplegia versus paraplegia), requiring additional treatments outside of their initial hospital admission [36]. Previous research has shown that patients with cervical spinal cord injuries were more likely to have had higher rates of complications and readmissions to the hospital, which likely would increase healthcare utilization and require more frequent visits to manage these problems [36–39]. In the long term, patients who had cervical injuries resulting in tetraplegia were more likely to have increased healthcare needs, including more intensive physical therapy, assistive devices, and caregiver needs, which led to higher healthcare resource utilization and spending [40].

Important for this study, this paper identified that where a patient resides has significant implications for accessing health care. Rural communities often have reduced access to healthcare providers and specialists, with a significant issue with access to PCP in the Canadian context [41]. Studies have shown that TSCI patients who reside further from spinal cord injury treatment centers were less likely to access specialized outpatient services for spinal cord injury [42]. This lack of access to care has been shown to be associated with being twice as likely to be admitted to the hospital for TSCI-related complications [43]. However, while relocation to an urban area may improve access to specialized care, it has also been associated with higher rates of depression and mental health concerns, likely due to a lack of social support from known environments [44]. Moreover, rural community networks may provide better socio-emotional support for patients with TSCI and have been associated with improved mental and physical health compared to those residing in urban communities [45, 46]. The advent of virtual care has been used to enable interactions with specialists working primarily in urban centres to provide treatment plans for TSCI-related complications and has been met with a positive response from these patients [47]. Further, there has been the implementation of workshops aimed at clinicians who work in rural communities to improve care of those with TSCI, which have demonstrated improved patient outcomes [48].

This study represented a large population-level retrospective study of physician service utilization using healthcare administrative data. The findings were representative of a single-payer healthcare system and utilized validated cohorts with a large sample size. However, there are several limitations to the study design. First, while this study evaluated the number of visits to a physician during the inclusion period, it did not characterize the nature or reason for the consultation. Second, many rehabilitation services delivered in the community and community-based home care may not have been publicly insured and not observable in this study. The services provided were also not evaluated to determine whether the consultations were meeting the patients’ needs or associated with patient outcomes. Next, the cohort was created based on hospital administrative data’s diagnostic codes. These codes are entered through hospital record review, but may contain errors and inaccuracies in reporting. The extent of neurological injury could also not be ascertained using these population-based databases. Finally, this study included data from the COVID-19 pandemic where changes in the delivery of care could have influenced the rates of healthcare utilization. However, studies have demonstrated that although in-person visits dramatically declined during the pandemic, the adoption of virtual care maintained steady rates of utilization for primary care, especially in Canada where the quick adoption of virtual care occurred [49]. This study included both virtual and in-person appointments in its analysis.

## Implications for care and policy

This study identified that patients with TSCI are frequent users of healthcare resources, with the most significant being primary care. This study builds upon previous knowledge in that patients who access services more are those who have cervical spinal cord injuries, likely worse neurological function, and those who live within urban centers. Future systems should be put in place to allow for improved access to care for patients with TSCI, with not just a focus on improving visit rates, but more importantly, the quality of care provided. PCPs should be provided with adequate resources and care guidelines for people with TSCI to help manage complex issues and ensure appropriate communication with specialists to ensure continuity of care. The Spinal Cord Injury Research Evidence (SCIRE) Professional group have established several guidelines and provides clinical updates specifically for PCP to help manage TSCI in the community [50]. Ideally, patients should be matched to a PCP with a special interest in TSCI and prior to discharge from hospital.

Priority must be given to the establishment of multidisciplinary teams of PCPs, specialists, and allied health services, which have been shown to improve outcomes and streamline care [34, 51]. Research and quality improvement initiatives should be aimed at improving efficiency and optimizing outcomes in TSCI by collaborating with specialized spinal cord injury centres and fostering relationships that span into community care [52]. Multidisciplinary team management of these patients in the community setting will be instrumental in assessing and providing optimal usage of health care resources, education for PCP, and help to improve communication amongst professionals to improve care. Finally, with the development and implementation of these multidisciplinary teams, research can determine which services are necessary and improve outcomes, reducing unnecessary medical encounters and healthcare spending.

## Conclusions

Among patients with TSCI, PCPs were found to be the primary contact with the healthcare system after their initial injury. Latent class analysis determined four distinct typologies of TSCI healthcare utilization, with cervical injury and urban residence being identified as the key components. Further research is needed to determine the differences in patterns of utilization based on extent of neurological injury and to evaluate whether multidisciplinary teams can improve access to care for those with TSCI.

## Supplementary Information

Below is the link to the electronic supplementary material.

Supplementary Material 1

## Data Availability

This study utilized discharge administrative data from Population Data B.C. for analysis. The data was obtained upon request from 2001 to 2021. Data use agreements prohibit sharing. Access to data provided by the Data Stewards is subject to approval, but can be requested for research projects through the Data Stewards or their designated service providers. The following data sets were used in this study: Discharge Abstract Database (DAD), Consolidation file (includes demographics, registry, and census geodata), B.C. Provincial Vital Statistics, the National Ambulatory Care Reporting System (NACRS), and the Medical Services Plan (MSP) Payment Information File, which includes physician billing data. You can find further information regarding these data sets by visiting the PopData project webpage at: [https://my.popdata.bc.ca/project_listings/15-119/collection_approval_dates] (https://my.popdata.bc.ca/project_listings/15-119/collection_approval_dates).

## Abbreviations

TSCI: Traumatic Spinal Cord Injury
PCP: Primary Care Provider
ED: Emergency Department
B.C.: British Columbia
DAD: Health Information Discharge Abstract Database
NACRS: The National Ambulatory Care Reporting System
MSP: The Medical Services Plan
ICD: International Classification of Diseases
ISNCSCI: International Standards for Neurological Classification of Spinal Cord Injury
CCI: Charlson Comorbidity Index
TBI: Traumatic Brain Injury
ISS: Injury Severity Score
QAIPPE: Quintile of Adjusted Income Per Person Equivalent
SCIRE: Spinal Cord Injury Research Evidence

## References

[1] Barbiellini Amidei C, Salmaso L, Bellio S, Saia M. Epidemiology of traumatic spinal cord injury: a large population-based study. Spinal Cord. 2022;60:812–9. 10.1038/s41393-022-00795-w. 35396455 10.1038/s41393-022-00795-w PMC8990493

[2] Zarmer L, Khan M, Islat G, Alameddin H, Massey M, Chaudhry R. Traumatic spinal cord injury: review of the literature. J Clin Med. 2025;14. 10.3390/jcm14113649. 10.3390/jcm14113649 PMC12155255 40507410

[3] Salaheen Z, Hejrati N, Wong IHY, Jiang F, Fehlings MG. Complications and adverse events following traumatic spinal cord injury. Neural repair and regeneration after spinal cord injury and spine trauma. Elsevier; 2022. pp. 385–99. 10.1016/B978-0-12-819835-3.00002-2.

[4] Lu Y, Shang Z, Zhang W, Pang M, Hu X, Dai Y, et al. Global incidence and characteristics of spinal cord injury since 2000–2021: a systematic review and meta-analysis. BMC Med. 2024;22:285. 10.1186/s12916-024-03514-9. 38972971 10.1186/s12916-024-03514-9 PMC11229207

[5] Lee BB, Cripps RA, Fitzharris M, Wing PC. The global map for traumatic spinal cord injury epidemiology: update 2011, global incidence rate. Spinal Cord. 2014;52:110–6. 10.1038/sc.2012.158. 23439068 10.1038/sc.2012.158

[6] Dryden DM, Saunders LD, Rowe BH, May LA, Yiannakoulias N, Svenson LW, et al. Utilization of health services following spinal cord injury: A 6-year follow-up study. Spinal Cord. 2004;42:513–25. 10.1038/sj.sc.3101629. 15249928 10.1038/sj.sc.3101629

[7] Krueger H, Noonan VK, Trenaman LM, Joshi P, Rivers CS. The economic burden of traumatic spinal cord injury in Canada. Chronic Dis Inj Can. 2013;33:113–22. 23735450

[8] Merritt CH, Taylor MA, Yelton CJ, Ray SK. Economic impact of traumatic spinal cord injuries in the United States. Neuroimmunol Neuroinflamm. 2019;2019. 10.20517/2347-8659.2019.15. 10.20517/2347-8659.2019.15 PMC8052100 33869674

[9] Pickett GE, Campos-Benitez M, Keller JL, Duggal N. Epidemiology of traumatic spinal cord injury in Canada. Spine (Phila Pa 1976). 2006;31:799–805. 16582854 10.1097/01.brs.0000207258.80129.03

[10] Bruggink C, van de Ree CLP, van Ditshuizen J, Polinder-Bos HA, Oner FC, Reijman M, et al. Increased incidence of traumatic spinal injury in patients aged 65 years and older in the Netherlands. Eur Spine J. 2024. 10.1007/s00586-024-08310-w. 38836903 10.1007/s00586-024-08310-w

[11] Chamberlain JD, Deriaz O, Hund-Georgiadis M, Meier S, Scheel-Sailer A, Schubert M, et al. Epidemiology and contemporary risk profile of traumatic spinal cord injury in Switzerland. Inj Epidemiol. 2015;2. 10.1186/s40621-015-0061-4. 10.1186/s40621-015-0061-4 PMC4630259 26550554

[12] Kannus P, Palvanen M, Niemi S, Parkkari J. Alarming rise in the number and incidence of Fall-Induced cervical spine injuries among older adults. J Gerontol Biol Sci Med Sci. 2007;62:180–3. 10.1093/gerona/62.2.180. 10.1093/gerona/62.2.180 17339643

[13] Trezzini B, Brach M, Post M, Gemperli A. Prevalence of and factors associated with expressed and unmet service needs reported by persons with spinal cord injury living in the community. Spinal Cord. 2019;57:490–500. 10.1038/s41393-019-0243-y. 30696925 10.1038/s41393-019-0243-y

[14] Parent S, Barchi S, LeBreton M, Casha S, Fehlings MG. The impact of specialized centers of care for spinal cord injury on length of stay, complications, and mortality: A systematic review of the literature. J Neurotrauma. 2011;28:1363–70. 10.1089/neu.2009.1151. 21410318 10.1089/neu.2009.1151 PMC3143414

[15] Sharwood LN, Stanford R, Middleton JW, Burns B, Joseph A, Flower O, et al. Improving care standards for patients with spinal trauma combining a modified e-Delphi process and stakeholder interviews: a study protocol. BMJ Open. 2017;7:e012377. 10.1136/bmjopen-2016-012377. 28104707 10.1136/bmjopen-2016-012377 PMC5253580

[16] Wiest MJ, Gargaro J, Bayley MT. What is the pathway to the best model of care for traumatic spinal cord injury? Evidence-Based guidance. Top Spinal Cord Inj Rehabil. 2023;29:103–11. 10.46292/sci23-00059S. 38174142 10.46292/sci23-00059S PMC10759857

[17] Wang TY, Park C, Zhang H, Rahimpour S, Murphy KR, Goodwin CR, et al. Management of acute traumatic spinal cord injury: a review of the literature. Front Surg. 2021;8. 10.3389/fsurg.2021.698736. 10.3389/fsurg.2021.698736 PMC8710452 34966774

[18] Ark TK, Kesselring S, Hills B, McGrail KM, Population Data BC. Supporting population data science in British Columbia. Int J Popul Data Sci. 2020;4. 10.23889/ijpds.v5i1.1133. 10.23889/ijpds.v4i2.1133 PMC7480325 32935036

[19] Canadian Institute for Health Information. Discharge Abstracts Database (Hospital Separations) data set: V2. Population Data BC. 2019. https://www.popdata.bc.ca/data/health/dad. Accessed 20 Mar 2024.

[20] British Columbia Ministry of Health. Vital Events Deaths. V2. Population Data BC. 2019. https://www.popdata.bc.ca/data/demographic/vs_deaths. Accessed 20 Mar 2024.

[21] British Columbia Ministry of Health. Medical Services Plan (MSP) Payment Information File. V2. Population Data BC. 2019. https://www.popdata.bc.ca/node/677. Accessed 20 Mar 2024.

[22] British Columbia Ministry of Health. Central demographics file (MSP registration and premium Billings, client roster and census Geodata)/Consolidation file (MSP registration and premium billing) data set. Popul Data BC. 2019.

[23] British Columbia Ministry of Health. National Ambulatory Care Reporting System data set. Population Data BC. 2019. https://www.popdata.bc.ca/data/health/nacrs. Accessed 15 Jul 2025.

[24] Noonan VK, Jaglal SB, Humphreys S, Cronin S, Waheed Z, Fallah N, et al. Top Spinal Cord Inj Rehabil. 2020;26:232–42. 10.46292/SCI20-00016. Linking Spinal Cord Injury Data Sets to Describe the Patient Journey Following Injury: A Protocol. 10.46292/sci20-00016 PMC7831289 33536728

[25] Osler T, Rutledge R, Deis J, Bedrick E. ICISS: an international classification of disease-9 based injury severity score. J Trauma. 1996;41:380–8. 10.1097/00005373-199609000-00002. 8810953 10.1097/00005373-199609000-00002

[26] Ontario Agency for Health Protection and Promotion. Summary Measures of Socioeconomic Inequalities in Health. Toronto, ON. 2013.

[27] Muthén L, Muthén B. Mplus User’s Guide. Eighth Edition. Los Angeles, CA. 2007.

[28] Milligan J, Lee J, Hillier LM, Slonim K, Craven C. Improving primary care for persons with spinal cord injury: development of a toolkit to guide care. J Spinal Cord Med. 2020;43:364–73. 10.1080/10790268.2018.1468584. 29733260 10.1080/10790268.2018.1468584 PMC7241547

[29] Ho CH. Primary care for persons with spinal cord injury — not a novel Idea but still under-developed. J Spinal Cord Med. 2016;39:500–3. 10.1080/10790268.2016.1182696. 27463240 10.1080/10790268.2016.1182696 PMC5020592

[30] Obembe AO, Simpson LA, Sakakibara BM, Eng JJ. Healthcare utilization after stroke in Canada- a population based study. BMC Health Serv Res. 2019;19:192. 10.1186/s12913-019-4020-6. 30917828 10.1186/s12913-019-4020-6 PMC6438024

[31] Laursen B, Helweg-Larsen K. Health service use in adults 20–64 years with traumatic brain injury, spinal cord injury or pelvic fracture. A cohort study with 9-year follow-up. BMJ Open. 2012;2. 10.1136/BMJOPEN-2012-001521. 10.1136/bmjopen-2012-001521 PMC3488711 23103605

[32] Noreau L, Noonan VK, Cobb J, Leblond J, Dumont FS. Spinal cord injury community survey: A National, comprehensive study to portray the lives of Canadians with spinal cord injury. Top Spinal Cord Inj Rehabil. 2014;20:249–64. 10.1310/sci2003-249. 25477739 10.1310/sci2004-249 PMC4252126

[33] Health Quality Ontario. Connecting the Dots for patients: family doctors’ views on coordinating patient care in ontario’s health system. Toronto; 2016.

[34] Ho C, Atchison K, Noonan VK, McKenzie N, Cadel L, Ganshorn H, et al. Models of care delivery from rehabilitation to community for spinal cord injury: A scoping review. J Neurotrauma. 2021;38:677–97. 10.1089/NEU.2020.7396. 33191849 10.1089/neu.2020.7396

[35] Nas K, Yazmalar L, Şah V, Aydin A, Öneş K. Rehabilitation of spinal cord injuries. World J Orthop. 2015;6:8–16. 10.5312/wjo.v6.i1.8. 25621206 10.5312/wjo.v6.i1.8 PMC4303793

[36] Skelton F, Hoffman JM, Reyes M, Burns SP. Examining health-care utilization in the first year following spinal cord injury. J Spinal Cord Med. 2015;38:690–5. 10.1179/2045772314Y.0000000269. 25299152 10.1179/2045772314Y.0000000269 PMC4725802

[37] Savic G, Short D, Weitzenkamp D, Charlifue S, Gardner B. Hospital readmissions in people with chronic spinal cord injury. Spinal Cord. 2000;38:371–7. 10.1038/sj.sc.3101019. 10889566 10.1038/sj.sc.3101019

[38] Gemperli A, Brach M, Debecker I, Eriks-Hoogland I, Scheel-Sailer A, Ronca E. Utilization of health care providers by individuals with chronic spinal cord injury. Spinal Cord. 2021;59:373–80. 10.1038/s41393-021-00615-7. 33597748 10.1038/s41393-021-00615-7

[39] Sikka S, Callender L, Driver S, Bennett M, Reynolds M, Hamilton R, et al. Healthcare utilization following spinal cord injury: objective findings from a regional hospital registry. J Spinal Cord Med. 2019;42:194–200. 10.1080/10790268.2018.1505330. 30277845 10.1080/10790268.2018.1505330 PMC6419654

[40] French DD, Campbell RR, Sabharwal S, Nelson AL, Palacios PA, Gavin-Dreschnack D. Health care costs for patients with chronic spinal cord injury in the veterans health administration. J Spinal Cord Med. 2007;30:477–81. 10.1080/10790268.2007.11754581. 18092564 10.1080/10790268.2007.11754581 PMC2141733

[41] Chen X, Orom H, Hay JL, Waters EA, Schofield E, Li Y, et al. Differences in rural and urban health information access and use. J Rural Health. 2019;35:405–17. 10.1111/jrh.12335. 30444935 10.1111/jrh.12335 PMC6522336

[42] Bell N, Kidanie T, Cai B, Krause JS. Geographic variation in outpatient health care service utilization after spinal cord injury. Arch Phys Med Rehabil. 2017;98:341–6. 10.1016/j.apmr.2016.09.130. 27984029 10.1016/j.apmr.2016.09.130

[43] Jaglal SB, Munce SEP, Guilcher SJ, Couris CM, Fung K, Craven BC, et al. Health system factors associated with rehospitalizations after traumatic spinal cord injury: a population-based study. Spinal Cord. 2009;47:604–9. 10.1038/sc.2009.9. 19274059 10.1038/sc.2009.9

[44] Glennie RA, Batke J, Fallah N, Cheng CL, Rivers CS, Noonan VK, et al. Rural and urban living in persons with spinal cord injury and comparing environmental Barriers, their Health, and Quality-of-Life outcomes. J Neurotrauma. 2017;34:2877–82. 10.1089/neu.2016.4931. 28462633 10.1089/neu.2016.4931 PMC5653139

[45] Whelan A, McVeigh S, Barker P, Glennie A, Wang D, Chen M, et al. The effect of rurality and distance from care on health outcomes, environmental barriers, and healthcare utilization patterns in persons with traumatic spinal cord injury. Spinal Cord. 2023;61:399–408. 10.1038/s41393-023-00898-y. 37169867 10.1038/s41393-023-00898-y PMC10173934

[46] Goodridge D, Rogers M, Klassen L, Jeffery B, Knox K, Rohatinsky N, et al. Access to health and support services: perspectives of people living with a long-term traumatic spinal cord injury in rural and urban areas. Disabil Rehabil. 2015;37:1401–10. 10.3109/09638288.2014.972593. 25332089 10.3109/09638288.2014.972593

[47] van de Pol E, Lucas K, Geraghty T, Pershouse K, Harding S, Atresh S, et al. The delivery of specialist spinal cord injury services in Queensland and the potential for telehealth. BMC Health Serv Res. 2015;16:29. 10.1186/s12913-016-1256-2. 10.1186/s12913-016-1256-2 PMC4727259 26810738

[48] Prins H, Donia S, Rockall S, Hektner J, Hawes S, Laskin JJ, et al. Implementing lived experience workshops in regional areas of British Columbia to enhance clinicians’ confidence in spinal cord injury care: an evaluation. Healthcare. 2024;12:731. 10.3390/healthcare12070731. 38610153 10.3390/healthcare12070731 PMC11011360

[49] Tu K, Sarkadi Kristiansson R, Gronsbell J, De Lusignan S, Flottorp S, Goh LH, et al. Changes in primary care visits arising from the COVID-19 pandemic: an international comparative study by the international consortium of primary care big data researchers (INTRePID). BMJ Open. 2022;12. 10.1136/BMJOPEN-2021-059130. 10.1136/bmjopen-2021-059130 PMC9086267 35534063

[50] Spinal Cord Injury Research Evidence. Primary Care and Spinal Cord Injury. https://scireproject.com/primary-care/. Accessed 10 Sep 2025.

[51] Jones ML, Gassaway J, Sweatman WM. Peer mentoring reduces unplanned readmissions and improves self-efficacy following inpatient rehabilitation for individuals with spinal cord injury. J Spinal Cord Med. 2021;44:383–91. 10.1080/10790268.2019.1645407. 31403374 10.1080/10790268.2019.1645407 PMC8081317

[52] Harnett A, Bateman A, Mcintyre A, Parikh R, Middleton J, Arora M, et al. Spinal Cord Injury Rehabilitation Practices. 2022.

